# Ufmylation System: Novel Control of GPCR Biosynthetic Trafficking and Therapeutic Targets

**DOI:** 10.3390/cells15211988

**Published:** 2026-10-31

**Authors:** Xin Xu, Guangyu Wu

**Affiliations:** Department of Pharmacology and Toxicology, Medical College of Georgia, Augusta University, Augusta, GA 30912, USA

**Keywords:** COPII vesicle, ER export, G protein-coupled receptor, maturation, trafficking, ufmylation

## Abstract

G protein-coupled receptors (GPCRs) constitute the largest family of membrane receptors that regulate a vast variety of cellular functions and serve as important therapeutic targets. A fundamental but poorly addressed question in GPCR biology is how individual receptors are efficiently delivered to their functional destinations following synthesis in the endoplasmic reticulum (ER). Ufmylation is a ubiquitination-like post-translational modification in which ubiquitin-fold modifier 1 is conjugated to target proteins. Compared with ubiquitination, the physiological and pathological significance of ufmylation is much less defined. Recent studies, however, have revealed crucial functions of the ufmylation system in maintaining ER homeostasis and directing GPCR export from the ER. In this review, we succinctly summarize recent advances in understanding the ufmylation system, discuss its role in the biosynthetic transport and segregation of nascent GPCRs at ER-derived COPII vesicles, and explore the therapeutic potential of targeting ufmylation-mediated GPCR trafficking.

## 1. Ufmylation System and Its Functions

### 1.1. Ufmylation

**Figure 1:**
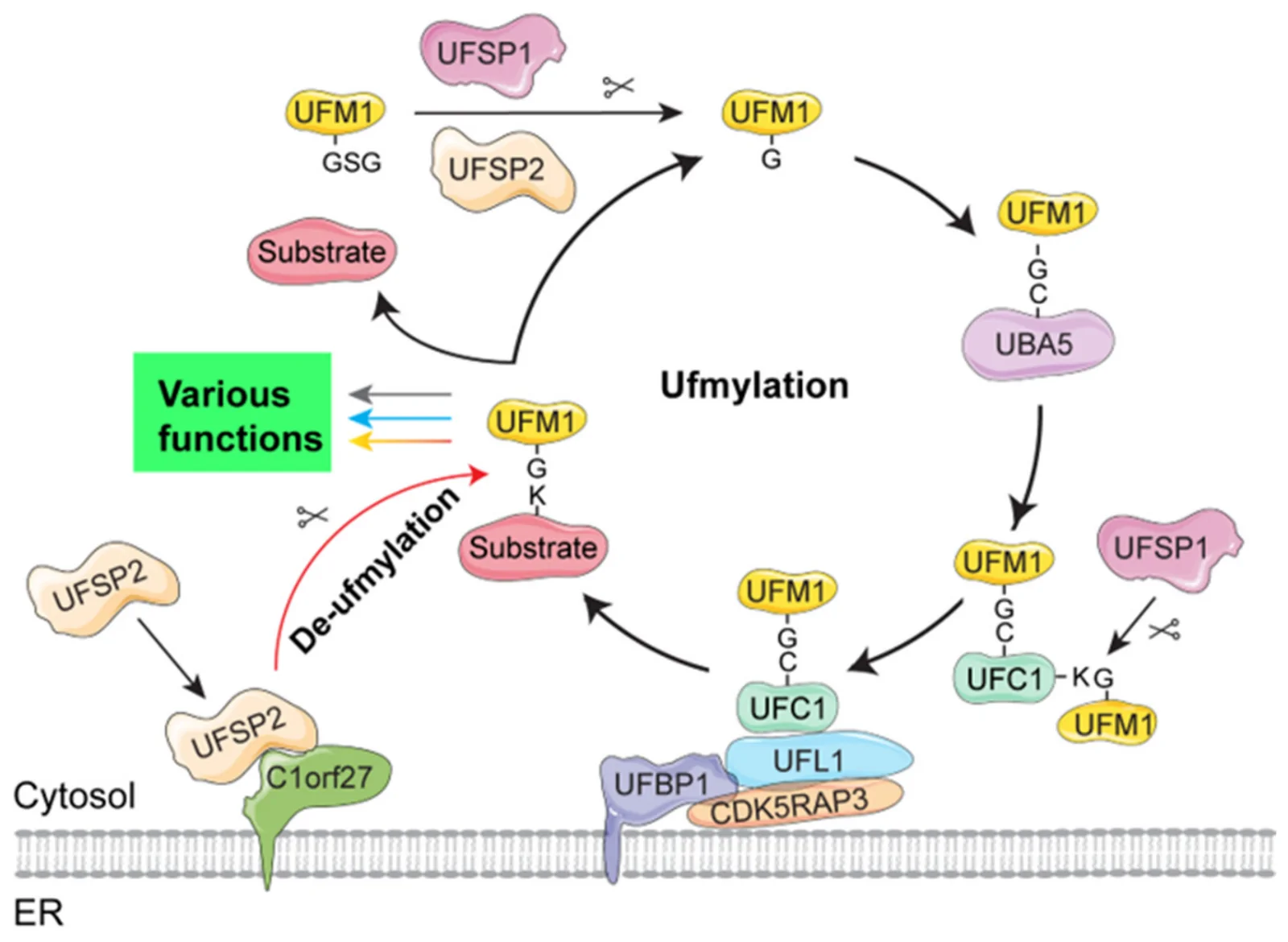
Schematic overview of the ufmylation cascade. UFSP1 and UFSP2 cleave UFM1 precursor to form mature UFM1. Following activation by the E1 UBA5, UFM1 is transferred sequentially to the E2 UFC1, the E3 UFL1 complex, and specific substrates. UFSP2 is responsible for removing UFM1 from ufmylated substrates. Two multi-protein complex, C1orf27-UFSP2 and UFBP1-UFL1-CDK5RAP3, anchor the ufmylation system at the ER (see text for detail).

Ufmylation is a reversible post-translational modification in which ubiquitin-fold modifier 1 (UFM1) is covalently conjugated to lysine (Lys) residues on target proteins through a three-enzyme cascade involving the E1 UFM1 activation enzyme 5 (UBA5), the E2 UFM1-conjugating enzyme 1 (UFC1), and the E3 UFM1-specific ligase 1 (UFL1). In this system, UFM1 is synthesized in the cell as an inactive precursor (pro-UFM1) containing 85 amino acid residues. UFM1-specific proteases (UFSPs) recognize pro-UFM1 and cleave its C-terminal two amino acid residues to expose the Gly83 residue, forming mature UFM1 which can proceed to the ligation reaction [1,2,3,4]. Mature UFM1 is activated by UBA5 via the formation of a thioester bond between the exposed Gly83 of UFM1 and the catalytic Cys250 of UBA5 [5,6,7,8]. The activated UFM1 is then transferred to UFC1 via the formation of a thioester bond with the catalytic Cys116 of UFC1 [6,9]. The final transfer of UFM1 to the Lys residues of specific target proteins is mediated via a scaffold-type E3 complex consisting of UFL1 and two adaptor proteins: UFM1-binding protein 1 (UFBP1, also known as DDRGK1 and C20orf116) and CDK5 regulatory subunit-associated protein 3 (CDK5RAP3). The interaction between UFC1 and UFL1 facilitates the transfer of the UFM1-UFC1 intermediate to the E3 complex, promoting the aminolysis of the UFM1-UFC1 thioester bond and leading to the exposure of UFM1 Gly83, which is then covalently conjugated to specific Lys residues of the target substrates [10,11,12,13] (Figure 1).

### 1.2. De-Ufmylation

Two UFSPs are dedicated to the ufmylation pathway: UFSP1 and UFSP2. They share an evolutionarily conserved, papain-like catalytic domain found in a large family of cysteine proteases. UFSP1 is a cytosolic protein mainly responsible for pro-UFM1 maturation and the removal of the UFM1 modification at Lys122 from UFC1 which relieves its inhibition [3,4]. Conversely, UFSP2 localizes predominantly at the ER membrane and serves as the primary protease responsible for removing UFM1 from ufmylated proteins, particularly ER-associated substrates such as RPL26, a component of the 60S ribosomal subunit (Figure 1) [14].

### 1.3. Substrates

Although ufmylation was discovered in 2004 [1], only a few UFM1 substrates were identified prior to 2020. UFBP1, an ER membrane protein, was the first identified UFM1 substrate [12,15,16] and its ufmylation occurs at Lys267 in humans (Lys268 in mice). Subsequent studies have demonstrated that, by virtue of its ability to assemble the E3 complex at the ER membrane, UFBP1 is a crucial regulator of the ufmylation system and is required for the ufmylation of numerous substrates. In the E3 complex, UFBP1 interacts with UFL1 to form the backbone of the E3 complex and enhances the activity of UFL1 to transfer UFM1 to downstream targets [10]. As such, the major function of UFBP1 in the ufmylation system differs from that of other UFM1 substrates. It is not just a substrate; it spatiotemporally regulates the activation of the cascade, particularly the E3 ligase UFL1 at the ER compartment, and subsequently controls the ufmylation of other downstream targets.

Research over the past six years has expanded the repertoire of UFM1 substrates (Table 1), which have significantly enhanced our understanding of biological functions of protein ufmylation. These substrates localize to different cellular compartments, such as the ER, cytosol, mitochondria, nucleus, cilia, and ribosomes. Among these substrates, RPL26 is the principal target of ufmylation. Its modification at Lys132 and Lys134 plays a critical role in regulating ER-ribosome-associated quality control (ER-RQC) [14,17].

### 1.4. Ufmylation and Its Cellular Functions at the ER

Two multi-protein complexes, UFL1-UFBP1-CDK5RAP3 and UFSP2-C1orf27, assemble at the ER membrane to dictate the activity of the ufmylation system on the cytosolic surface of the ER [68]. As discussed in Section 1.3, the UFL1-UFBP1-CDK5RAP3 complex controls both the activation and spatial localization of UFL1 at the ER. Similar to UFBP1, C1orf27 is an ER-anchored membrane protein whose interaction with UFSP2 regulates the localization of UFSP2 at the ER (Figure 1). In addition, immunoblotting revealed that while UFSP2 knockout clearly reduced C1orf27 expression, C1orf27 knockout exerted a much more profound inhibitory effect on UFSP2 expression in HEK293 cells [4]. These data suggest that the formation of the C1orf27-UFSP2 complex mutually stabilizes both proteins. As such, the molecular mechanisms and cellular functions of the ufmylation system at the ER membrane have been extensively studied.

The ufmylation system regulates a broad spectrum of cellular processes, including cell cycle progression, nuclear signaling, the DNA damage response, genomic integrity, cell development, and cell death [69,70,71,72,73,74]. At the ER level, ufmylation is well established as an important regulator of ER-RQC, ER-phagy, ER stress, and ER-associated protein degradation (ERAD) thereby playing a significant role in maintaining ER homeostasis [13,15,17,18,19,20,73,75,76]. These ER-associated functions have been thoroughly reviewed in several articles [77,78,79,80].

Recent studies have revealed that the ufmylation system can also control protein export from the ER. This includes the unconventional secretion of α-synuclein (a UFM1 substrate) [32], the secretion of lipoproteins [81], and the transport of selective G protein-coupled receptors (GPCRs) [82,83,84]. Here we discuss the specific functions of the ufmylation system in regulating the biosynthetic trafficking and sorting of GPCRs at ER-derived COPII vesicles.

## 2. The Role of the Ufmylation System in the Biosynthetic Trafficking of GPCRs

### 2.1. The Ufmylation Cascade Controls Nascent GPCR Export from the ER to the Golgi

Similar to other plasma membrane (PM) proteins, GPCRs are synthesized in the ER. Nascent receptors are co-translationally integrated into the ER membrane, where they undergo modification, folding, and assembly. Once they pass through the scrutiny of the ER-quality control system, they are exported from the ER and subsequently transported to the Golgi apparatus and the cell surface. Over the past few decades, numerous regulatory proteins [85,86,87,88,89,90,91,92,93,94,95] and structural determinants embedded within the receptors [96,97,98,99,100,101,102] have been identified as key regulators in receptor folding, maturation, assembly, COPII vesicle recruitment, microtubule-mediated transport, and cargo sorting. However, compared to the well-studied mechanisms of receptor internalization, how GPCRs target to the cell surface *en route* from the ER and traffic through the Golgi apparatus remains much less understood.

**Figure 2:**
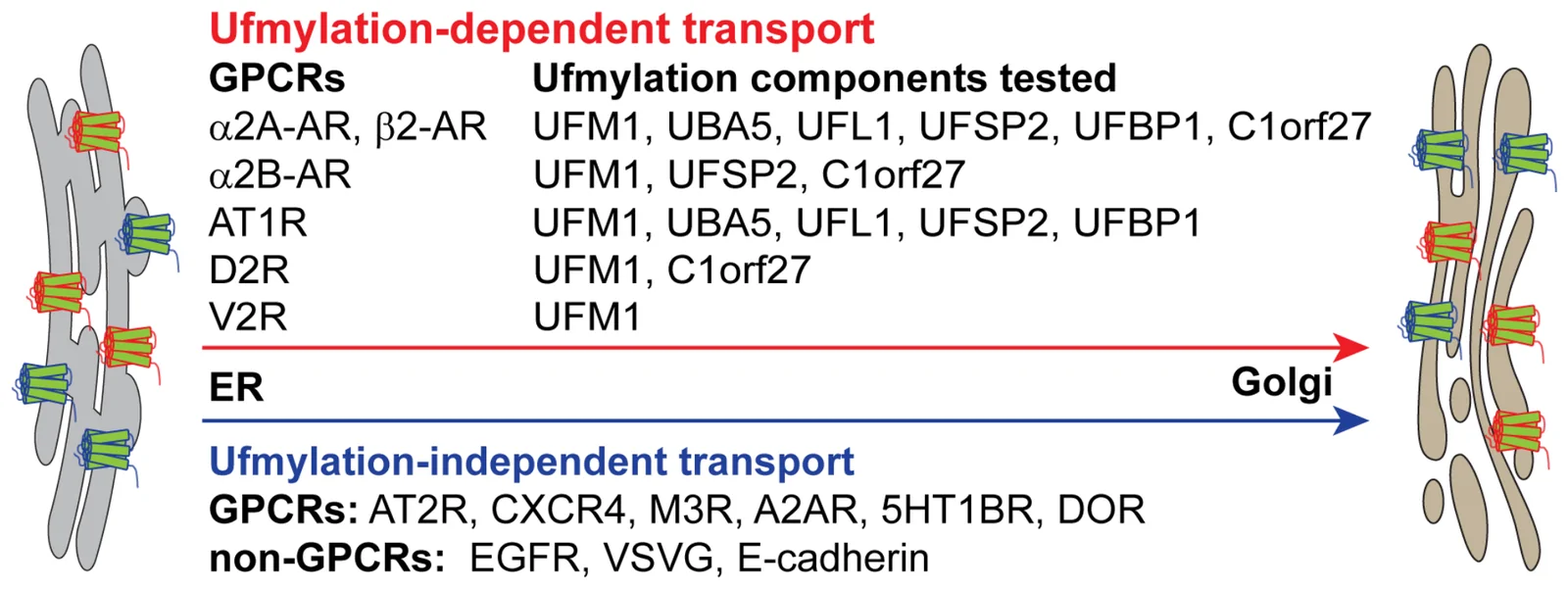
Ufmylation-dependent and -independent ER-to-Golgi transport of GPCRs and non-GPCR PM proteins. As demonstrated using RUSH assays in the cells depleted individual ufmylation components, GPCRs may use ufmylation-dependent and -independent pathways to transport from the ER to the Golgi, whereas all three non-GPCR plasma membrane proteins studied thus far use a ufmylation-independent route. α_2A_-AR, α_2A_-adrenergic receptor; AT1R and AT2R, angiotensin II type 1 and type 2 receptors; D2R, dopamine D2 receptor; V2R, vasopressin V2 receptor; M3R, muscarinic acetylcholine receptor type 3; A2AR, adenosine A2A receptor; 5HT1BR, 5-hydroxytryptamine receptor 1B; DOR, δ opioid receptor; EGFR, epidermal growth factor receptor; VSVG, vesicular stomatitis virus glycoprotein.

The functional role of the ufmylation system in GPCR biosynthesis was identified through a screening assay, where UFM1 depletion remarkably blocked the ER-to-Golgi transport of six out of twelve GPCRs studied, while having no effect on three non-GPCR PM proteins [82] (Figure 2). This function was further supported by findings from the individual depletion of UBA5, UFL1, UFSP2, and UFBP1. In these studies, the ER-to-Golgi transport was quantified using the retention using selective hooks (RUSH) assays, in which GPCRs were conjugated with a streptavidin-binding peptide (SBP), while the ER retention signal KDEL fused to streptavidin served as the hook to prevent the ER export of nascent receptors [103]. Receptor export from the ER was then synchronized by adding biotin to disrupt the interaction between SBP and streptavidin. Because the depletion of individual ligases, proteases, and regulators involved in the ufmylation pathway produced comparable inhibitory effects on the ER-to-Golgi transport of specific cargo GPCRs, the entire ufmylation system, encompassing both ufmylation and de-ufmylation processes, is vital for proper GPCR export [82,84].

Among the known UFM1 substrates, the roles of UFBP1 and RPL26 ufmylation have been evaluated using different strategies. The impact of UFBP1 ufmylation was demonstrated in rescue experiments using its ufmylation-deficient K267R mutant. Because the rescue efficiency of the K267R mutant is approximately 50% compared to its wild-type counterpart [82], the function of UFBP1 in GPCR transport is partially dependent on its own ufmylation. This is consistent with the primary function of UFBP1 in regulating the activation and localization of the E3 ligase UFL1 as discussed in Section 1.3. Conversely, RPL26 ufmylation is dispensable for GPCR trafficking, as revealed in ufmylation-deficient mutant knock-in cells [82]. In addition, defective receptor export is accompanied by neither unfolded protein response (UPR) nor receptor degradation, suggesting that the role of the ufmylation system in GPCR transport is mechanistically distinct from its established function in maintaining ER homeostasis [82].

### 2.2. Ufmylation Affects GPCR Recruitment to COPII Vesicles

**Figure 3:**
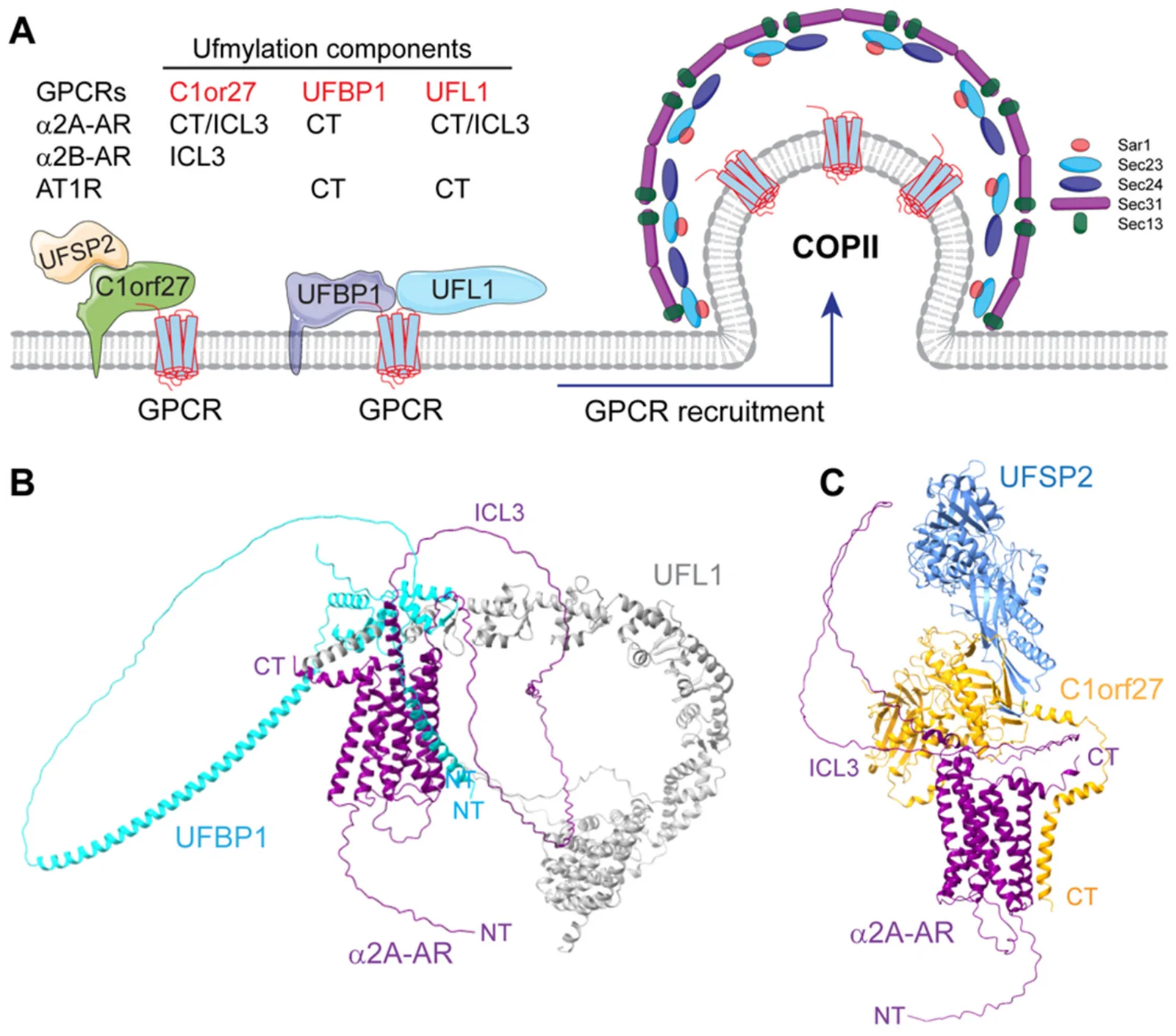
GPCR interaction with multiple components in the ufmylation system regulates their recruitment to COPII vesicles and subsequent export from the ER. (**A**) A model showing the possible function of two ternary complexes, GPCR-C1orf27-UFSP2 and GPCR-UFBP1-UFL1, formed at the ER in regulating cargo GPCR recruitment to COPII vesicles. α_2A_-AR and α_2B_-AR have been shown to interact with C1orf27 which interacts UFSP2, whereas α_2A_-AR and AT1R interact with both UFBP1 and UFL1 directly, forming two ternary complexes. (**B**,**C**) Structural prediction of the complexes α_2A_-AR-UFBP1-UFL1 (**B**) and α_2A_-AR-C1orf27-UFSP2 (**C**) by AlphaFold 3. NT, N-terminus; CT, C-terminus; ICL3, third intracellular loop.

COPII vesicles mediate the export of newly synthesized secretory and membrane proteins, as well as lipids, from the ER. COPII vesicle assembly occurs at ER exit sites (ERES) and is initiated by the ER recruitment and activation of Sar1 GTPase via the guanine nucleotide exchange factor Sec12. Activated Sar1 interacts with Sec23 and recruits the Sec23-Sec24 heterodimer to form the inner layer of COPII vesicles. The Sec23-Sec24 complex subsequently recruits the Sec13-Sec31 complex to compose the outer layer, which polymerizes the coat and drives the membrane curvature to bud the COPII vesicles (Figure 3A) [104,105,106]. For membrane cargo proteins, recognition and concentration into COPII vesicles are primarily dictated by direct interactions with Sec24, whereas soluble cargoes and certain membrane proteins lacking direct COPII-binding motifs rely on cargo-specific receptors [107,108].

Our recent studies have demonstrated that the ufmylation system acts at COPII vesicles to control GPCR export from the ER. All GPCRs studied thus far utilize COPII vesicles for their exit from the ER, as inhibiting Sar1 function to disrupt COPII vesicle formation almost completely abolishes their transport from the ER to the Golgi and the cell surface [82,109]. However, distinct GPCRs possess different capacities to concentrate within COPII vesicles, as revealed by using the RUSH system to anchor newly synthesized GPCRs in the ER [109]. In particular, angiotensin II type 1 and 2 receptors (AT1R and AT2R) and CXCR4 are actively recruited and highly concentrated in COPII vesicles [109]. Interestingly, although α_2B_-adrenergic receptor (α_2B_-AR) interacts with Sec24C and Sec24D via a triple-arginine (3R) motif located in its third intracellular loop (ICL3) [96] and β_2_-AR interacts with cornichon homolog 4 (CNIH4), a potential cargo transport receptor [110], neither is clearly concentrated at COPII vesicles when measured in RUSH assays [109]. These findings demonstrate that different GPCRs utilize distinct molecular mechanisms for targeting to COPII vesicles.

Ufmylation components, including UFBP1 and CDK5RAP3, have been identified at COPII vesicles by proteomics and immunoblotting following proximity biotinylation assays using the promiscuous biotin ligase BirA mutant R118G (BirA*) that catalyzes biotinylation of neighboring proteins [82,111,112]. More interestingly, BirA*-conjugated UFBP1 clearly biotinylated Sar1B, Sec24A, Sec24B, and Sec24D, but not Sec24C, suggesting that UFBP1 may localize to specific subsets of COPII vesicles. In addition, UFBP1 and UFL1 may physically associate with Sar1 [81]. Importantly, the depletion of ufmylation components markedly inhibits the biotinylation of all studied GPCRs by Sar1-BirA* without affecting overall COPII vesicle biogenesis [82]. Altogether, these studies support the notion that the ufmylation system enhances the COPII capture of nascent cargo GPCRs, thereby facilitating their export from the ER (Figure 3A).

### 2.3. GPCR Interaction with the UFBP1-UFL1 Complex Controls Their Export and Sorting at the ER

As the GPCR superfamily consists of over 800 members that share a conserved structural topology, how they are sorted from one another and from non-GPCR PM proteins during maturation processing following synthesis in the ER is an important question in studying GPCR trafficking. Previous studies suggest that different GPCRs, may utilize distinct biosynthetic transport pathways for their delivery to the cell surface [95,113,114,115], and several proteins capable of sorting different GPCRs, even very closely related members such as α_2A_-AR and α_2B_-AR, have been identified [113,114,116,117].

Because the depletion of ufmylation components selectively affects the ER export of only some, but not all, GPCRs or PM proteins, the ufmylation system appears to have the capacity to segregate different cargo proteins at the ER level. This sorting function is likely mediated by a direct interaction with cargo molecules. For instance, two ufmylation-dependent GPCRs, α_2A_-AR and AT1R, interact with UFBP1 and UFL1 (Figure 3A), whereas the ufmylation-independent AT2R does not [82]. Structural prediction by AlphaFold 3 suggests that α_2A_-AR may interact with both UFBP1 and UFL1 through its C-terminus (CT) and the ICL3 (Figure 3B). Furthermore, fusing to the AT1R CT, which binds UFBP1 and UFL1, to vesicular stomatitis virus glycoprotein (VSVG) effectively converts VSVG transport from ufmylation-independent to ufmylation-dependent. These data provide direct evidence indicating that these protein–protein interactions drive cargo separation. Therefore, physical associations between cargo GPCRs and ufmylation components may dictate cargo recruitment and selection at COPII vesicles.

### 2.4. The C1orf27-UFSP2 Complex Positions GPCRs for Ufmylation Regulation

As discussed in Section 1.4, C1orf27 and UFSP2 form a complex that anchors UFSP2, as well as the de-ufmylation machinery, at the ER. Similar to UFSP2 depletion, C1orf27 depletion markedly inhibits the de-ufmylation of specific UFM1 substrates [19,68], suggesting that the C1orf27-UFSP2 complex acts as a functional de-conjugating enzyme complex, analogous to the function of the E3 UFL1 complex in ufmylation. Mirroring their function in protein de-ufmylation, the depletion of C1orf27 and UFSP2, individually or in combination, produces similar inhibitory effects on the ER-to-Golgi transport of GPCRs, indicating that their actions in GPCR transport are cooperative rather than additive [83,84].

In addition to binding to UFSP2, C1orf27 directly interacts with cargo GPCRs, including α_2A_-AR and α_2B_-AR [83,84]. Although UFSP2 does not directly interact with GPCRs, it associates with the receptors via its interaction with C1orf27, leading to the formation of a linear ternary complex: GPCR-C1orf27-UFSP2 [83]. It is interesting to note that α_2A_-AR interaction with C1orf27 is mediated through both its CT and ICL3, whereas α_2B_-AR utilize only ICL3 to interact with C1orf27 [83]. AlphaFold modeling also suggests that while α_2A_-AR physically engages with C1orf27, it does not directly contact UFSP2 (Figure 3C).

The function of the C1orf27-UFSP2 complex in GPCR trafficking was initially identified in *C. elegans*. Odorant response abnormal 4 (ODR4) and ODR8 are *C. elegans* homologs of C1orf27 and UFSP2, respectively. Both proteins are essential for the trafficking of a subset of olfactory GPCRs, such as ODR10, to the cilia of olfactory neurons, and ODR4 directly interacts with ODR10 [118,119]. Subsequent studies establish that ODR4 physically interacts with and recruits ODR8 to the ER membrane to form a complex [119]. Altogether, these data demonstrate that GPCR export from the ER in humans and nematodes is controlled by parallel, evolutionarily conserved systems in which the protein trio of GPCRs/ODR10, C1orf27/ODR4 and UFSP2/ODR8 forms functional ternary complexes [83,119].

However, the requirement of catalytic activity of UFSP2/ODR8 in GPCR transport remains controversial between *C. elegans* and mammalian cells. In *C. elegans*, ODR8 regulates olfactory receptor transport independently of its enzymatic activity [119]. Conversely, in mammalian cells, catalytically inactive UFSP2 mutants retain their ability to be recruited to the ER by C1orf27 but are unable to rescue transport deficiencies in UFSP2 knockout cells. Furthermore, when expressed in wild-type cells, these inactive variants act as dominant-negative mutants inhibiting cargo GPCR export from the ER, which is likely mediated through their interactions with both C1orf27 and cargo GPCRs, forming an inactive ternary complex that blocks receptor export from the ER [83]. These data also suggest that the C1orf27-UFSP2 complex and nascent GPCRs assemble into a ternary complex at the ER, thereby positioning the receptors for ufmylation-dependent ER export [82,83].

Several potential mechanisms may explain the species-specific function of UFSP2/ODR8 in GPCR export from the ER. First, UFSP2/ODR8 may have distinct substrates whose ufmylation exerts divergent effects on GPCR transport in mammalian cells versus *C. elegans*. Second, the UFSP2/ODR8-C1orf27/ODR4 complex might function as a molecular chaperone that physically interacts with cargo GPCRs to facilitate their maturation. However, in mammalian system, the enzymatic activity of UFSP2 is required to target the ternary complex GPCR-C1orf27-UFSP2 to COPII vesicles as discussed above. Alternatively, this discrepancy may simply arise from the different experimental approaches used to monitor GPCR transport. While studies in *C. elegans* primarily characterize the steady-state localization of olfactory receptors at sensory cilia, studies in mammalian cells utilize RUSH assays to precisely quantify synchronized GPCR transport from the ER to the Golgi. Nevertheless, these findings indicate that olfactory and classic GPCRs utilize distinct regulatory mechanisms to govern their biosynthetic maturation.

## 3. Therapeutic Potential of Targeting GPCR Biosynthetic Trafficking and Ufmylation

For most GPCRs, the cell surface is their functional destination where they bind to extracellular ligands and drugs to initiate physiological and pathological responses. Several studies suggest that ER export is a rate-limiting step in GPCR maturation process and dictates the abundance of receptors at the cell surface which in turn governs the strength of receptor-mediated downstream cellular signaling [95,114,120]. Indeed, the dysregulation of GPCR biosynthetic transport, resulting in either enhancement or attenuation in the surface expression of functional receptors, is linked to the pathogenesis of a variety of human diseases [121]. Many of these diseases are caused by naturally occurring mutations that lead to receptor misfolding and inability to export from the ER. Classic examples include retinitis pigmentosa (associated with rhodopsin), X-linked nephrogenic diabetes insipidus (NDI) [vasopressin 2 receptor (V2R)], congenital hypogonadotropic hypogonadism (gonadotropin-releasing hormone receptor), hypogonadotropic hypogonadism (luteinizing hormone receptor), and morbid early onset obesity (melanocortin 3 and 4 receptors). As many of these misfolded receptors remain intrinsic activity once they reach the cell surface, there is immense interest in developing therapeutic strategies to rescue their ER export and restore their normal PM localization and function [122,123,124,125,126].

For example, more than 300 distinct mutations have been identified in V2R, a receptor whose surface transport is regulated by the ufmylation system (Figure 2) [82]. The vast majority of these mutations cause X-linked NDI, as the mutated receptors are trapped within the ER which prevents the receptors from responding to antidiuretic hormones, causing the kidneys to lose the ability to concentrate urine. Interestingly, several cell-permeable, non-peptide V2R antagonists, such as tolvaptan (OPC-41061), satavaptan (SR121463), relcovaptan (SR49059), and VPA-985, act as pharmacological chaperones (pharmacoperones); they enhance the surface expression of some V2R mutants and restore their abilities to bind ligands and activate cellular response [124,127].

The contributions of the ufmylation system to human diseases have been extensively studied in animal models through the genetic depletion of core ufmylation components (e.g., UFM1, UBA5, UFC1, UFL1, UFSP2), regulators (e.g., UFBP1 and CDK5RAP3), and specific UFM1 substrates (Table 1). In addition, ufmylation-deficient mutant knock-in strategies have been utilized to define the pathological roles of individual UFM1 substrates [17,128]. These studies have clearly demonstrated that the ufmylation system is implicated in a broad spectrum of human diseases as previously reviewed [46,74,129,130,131], implicating that it is a therapeutic target. Indeed, several small-molecule pharmacological inhibitors of UBA5, such as DKM 2-93, have already been developed and evaluated for cancer treatment in preclinic animal models [63,132].

Consistent with the primary function of the ufmylation system in maintaining the ER homeostasis, a number of ufmylation-related diseases, including type 2 diabetes (associated with UFM1, UFL1 and UFBP1) [133,134], exocrine pancreatic disease (UFL1) [135], non-alcoholic fatty liver disease (UFBP1 and P4HB) [15,23], heart disease (UFL1 and UFBP1) [136,137], inflammatory bowel diseases (UFL1 and UFBP1) [138], and kidney disease (UFL1 and UFBP1) [139,140], likely result from chronic disruptions in ER hemostasis. Another major feature of ufmylation-associated pathologies is linked to neurodevelopmental failure and accelerated neurodegeneration which are driven by naturally occurring mutations in core ufmylation components, aberrant proteostasis and/or the accumulation of neurotoxic proteins, such as α-synuclein and Tau [32,141]. These conditions include severe pediatric neurodevelopmental disorders (UFSP2) [142], epileptic encephalopathy (UBA5) [143,144], microcephaly (UFL1) [69], autosomal recessive cerebellar ataxia (UBA5) [145], Alzheimer’s disease (UFSP2, UBA5 and Tau) [141,146], and Parkinson’s disease (UFBP1, UFM1, UBA5 and α-synuclein) [32,147].

Although these studies highlight the crucial roles of the ufmylation system in human pathology, the detailed underlying mechanisms remain poorly defined. Given that GPCRs are vital regulators of almost every cellular function under both physiological and pathological conditions, it is possible that these diseases are, in part, attributable to abnormal GPCR trafficking and signaling caused by ufmylation system defects. Therefore, thoroughly characterizing the functional intersections of ufmylation, GPCR trafficking, and downstream signaling will provide an important foundation for designing next generation therapeutics.

## 4. Concluding Remarks

Over the past several years, great progress has been made in identifying ufmylation substrates and defining the roles of ufmylation in maintaining the ER homeostasis, protein secretion, and export trafficking. The function of the ufmylation cascade in regulating the ER export of GPCRs is likely mediated by facilitating their recruitment to COPII vesicles through two distinct complexes assembled on the ER membrane: UFBP1-UFL1 and C1orf27-UFSP2, both of which directly interact with cargo GPCRs [82,83,84] (Figure 3). These multi-protein complexes provide molecular platforms that coordinate cargo recognition and direct the receptors to the ufmylation machinery at the ER membrane. In addition, many GPCRs are known to undergo homodimerization in the ER [148], and C1orf27 has been shown to interact with UFBP1 [68]. This suggests that multiple ufmylation components (such as C1orf27, UFSP2, UFBP1, UFL1, and CDK5RAP3) and cargo GPCRs may assemble into a much larger complex to tightly control GPCR export from the ER. Thus, the role of the ufmylation system in mediating GPCR anterograde trafficking is likely directed by a sophisticated mechanism involving multiple protein–protein interactions and the spatiotemporal coordination of substrate ufmylation and de-ufmylation.

### Despite These Compelling Findings, Several Critical Questions Remain Unanswered

(1) Identification of key substrates: Because RPL26 ufmylation is dispensable for GPCR trafficking and UFBP1 function depends only partially on its ufmylation, the key UFM1 substrates that dictate GPCR export from the ER in a ufmylation-dependent manner remain to be discovered.
(2) Validation of endogenous GPCR trafficking: The functional role of the ufmylation system in regulating the anterograde transport of endogenous GPCRs in their native cellular environment needs to be firmly established. Most GPCR trafficking studies to date have been conducted using overexpression models, which may exceed the ability of endogenous transport machinery. However, monitoring the export kinetics of endogenous GPCRs at low expression levels along the secretory pathway remains a major technical challenge.
(3) Mechanisms of cargo selection: How the ufmylation system selectively recognizes its cargo GPCRs and segregates specific receptor members from one another, as well as from non-GPCR membrane proteins, remains a fundamental question in the field.

## Figures and Tables

**Table 1:** Ufmylation substrates.

Substrates	Localization	Ufmylation Sites	Associated Functions and Diseases	References
UFBP1	ER	K267	ER homoeostasis, metabolic diseases	[12,15,16]
CYB5R3	ER	K214	ER-phagy	[18]
RPN1	ER		ER-phagy	[19]
HRD1	ER	K610	ERAD	[20]
Fmo1	ER		Ferroptosis, liver injury	[21]
GRP78	ER	K294/296/352/353/370	ER homeostasis	[22]
P4HB	ER, mitochondria, cytosol	K69/114/130	ER stress, oxidative stress	[23]
RPL26	60S ribosome, cytosol	K132/134	ER-RQC	[14]
RPL10	60S ribosome, cytosol	K30/101	Cell proliferation, stemness	[24]
OFIP	Centrosome		Centrosomal localization	[25]
14-3-3ε	Cytosol		Antiviral response	[26]
NLRP3	Cytosol	K320/375/426/492	Inflammasome activation	[27]
ENO1	Cytosol	K285/420	Glycolysis, colorectal cancer	[28]
AKT1	Cytosol	K189/276/297	Lipid synthesis, breast cancer	[29,30]
PLAC8	Cytosol, PM	K103	Cell proliferation, immune responses	[31]
α-Synuclein	Cytosol, PM		α-Synuclein secretion	[32]
Eg5	Cytosol, mitotic spindle	K564/953	Mitosis, ciliary homeostasis	[33,34]
IFT88	Cytosol, cilium	K572	Ciliary homeostasis	[35]
MAVS	Mitochondria	K461	Inflammasome evasion	[36]
MRE11	Nucleus	K281/282	Genome integrity, telomere length	[37,38]
MCM5	Nucleus	K583	DNA replication	[39]
ERa	Nucleus	K171/180	Transactivation, breast cancer	[40]
Histone H4	Nucleus	K31	Genome integrity	[41]
PARP1	Nucleus	K548	DNA replication	[42]
BAP1	Nucleus	K51/61/187/205	Colorectal cancer	[43]
PTIP	Nucleus	K148/173/175/176	DNA replication	[44,45]
NONO	Nucleus	K198	Glycolysis, cell proliferation	[46]
EMD	Nucleus		Liver cancer	[47]
RNF20	Nucleus	K530/561/563/588	Prostate cancer	[48]
SREBP1	Nucleus		Lipid metabolism, cell proliferation	[49]
p53	Nucleus, cytosol	K351/357/370/373	Cell proliferation	[50]
ASC1	Nucleus, cytosol	K324/K25/K34/K67	Breast cancer	[51]
HIF1α	Nucleus, cytosol	K674/682/721	Hypoxic tumor growth	[52]
YAP	Nucleus, cytosol		Endothelial cell senescence	[53]
ArpC4	Nucleus, cytosol	K60/84/107/130/166	Cell migration	[54]
VCP	Nucleus, cytosol	K109	Autophagy	[55]
Ku70	Nucleus, cytosol	K526	Genome integrity	[56]
KEAP1	Nucleus, cytosol		Liver cancer	[57]
p62	Nucleus, cytosol	K420/435	Autophagy	[58]
STAT3	Nucleus, cytosol		Cartilage homeostasis	[59]
MT1M	Nucleus, cytosol	K31	NF-kB activity	[60]
TSC22D3	Nucleus, cytosol	K127	NF-kB activity	[60]
Pirin	Nucleus, cytosol		Ferroptosis	[61]
PRMT5	Nucleus, cytosol, Golgi		Tumor immune evasion	[62]
PD-L1	PM	K75/89/105/162/280/281	Tumor immune evasion	[63]
SLC7A11	PM		Ferroptosis, breast cancer	[64]
PD-1	PM	K210/233	T cell immune activity	[65]
PHLDA3	PM, cytosol	K51/106	Lung adenocarcinoma	[66]
ET-1			Melanin biosynthesis, pigmentation	[67]

CYB5R3, NADH-cytochrome B5 reductase 3; RPN1, ribophorin 1; HRD1, HMG-CoA reductase degradation protein; Fmo1, flavin containing dimethylaniline monooxygenase 1; GRP78, glucose-regulated protein 78; P4HB, prolyl 4-hydroxylase subunit β; RPL26, ribosomal protein L26; RPL10, ribosomal protein L10; NLRP3, pyrin domain-containing protein 3; ENO1, glycolytic enzyme enolase 1; AKT1, AKT serine/threonine kinase 1; MT1M, metallothionein-1M; TSC22D3, TSC22 domain family protein 3; PLAC8, placenta-specific gene 8 protein; IFT88, intraflagellar transport 88; MAVS, mitochondrial antiviral signaling protein; MRE11, double strand break repair nuclease MRE11; MCM5, minichromosome maintenance complex component 5; ERα, estrogen receptor α; PARP1, poly (ADP-Ribose) polymerase 1; BAP1, BRCA1-associated protein 1; PTIP, Pax2 transactivation domain interacting protein; NONO, non-POU-domain-containing octamer-binding protein; RNF20, ring finger protein 20; SREBP1, sterol regulatory element binding transcription factor 1; ASC1, activating signal co-integrator 1 complex subunit 1; HIF1α, hypoxia inducible factor 1 subunit α; YAP, hippo-Yes-associated protein; ArpC4, actin related protein 2/3 complex subunit 4; VCP, valosin containing protein; KEAP1, kelch-like ECH-associated protein 1; STAT3, signal transducer and activator of transcription 3; PRMT5, protein arginine methyltransferase 5; PD-L1, programmed cell death 1 ligand 1; SLC7A11, solute carrier family 7 member 11; PD-1, programmed cell death 1; PHLDA3, pleckstrin homology like domain family A member 3; ET-1, endothelin-1. ER, endoplasmic reticulum; PM, plasma membrane; ERAD, ER-associated protein degradation; ER-RQC, ER-ribosome-associated quality control.

## Data Availability

The data presented in this study are available from the corresponding author upon request.
